# Astroblastoma – a rare and challenging tumor: a case report and review of the literature

**DOI:** 10.1186/s13256-018-1623-1

**Published:** 2018-04-21

**Authors:** Nawal Hammas, Nadia Senhaji, My Youssef Alaoui Lamrani, Sanae Bennis, Elfaiz Mohamed Chaoui, Hind El Fatemi, Laila Chbani

**Affiliations:** 1 Department of Pathology, Hassan II University Hospital, 30000 Fez, Morocco; 2 Biomedical and Translational Research Laboratory, Faculty of Medicine and Pharmacy, Sidi Mohamed Ben Abdellah University, Fez, Morocco; 3 Bioactive Molecules Laboratory, Faculty of Science and Technology, Sidi Mohamed Ben Abdellah University, Fez, Morocco; 4 Department of Radiology, Hassan II University Hospital, Fez, Morocco; 5 Faculty of Medicine and Pharmacy, Sidi Mohamed Ben Abdellah University, Fez, Morocco; 6 Oncogenetic/pathology Unit, Hassan II University Hospital, Fez, Morocco; 7 Department of Neurosurgery, Hassan II University Hospital, Fez, Morocco

**Keywords:** Astroblastoma, Brain neoplasm, Histogenesis, Immunohistochemistry, Differential diagnosis

## Abstract

**Background:**

Astroblastoma is a controversial and an extremely rare central nervous system neoplasm. Although its histogenesis has been clarified recently, controversies exist regarding its cellular origin and validity as a distinct entity. Because of its extreme rarity and because its common features are shared with other glial neoplasms, this tumor is prone to misdiagnosis and remains challenging not only in terms of diagnosis and classification but also in the subsequent management. This case report describes a new case of astroblastoma. It discusses clinical, radiologic, pathological, and therapeutic features and differential diagnosis of this rare neoplasm, with a review of the recent literature.

**Case presentation:**

We report the case of an 8-year-old Moroccan girl who presented with a 1-year history of epileptic seizure, headache, and decreased visual acuity. Cranial magnetic resonance imaging revealed a right occipito-temporal mass. A tumor resection was performed and histological examination combined with immunohistochemical study confirmed the diagnosis of low-grade astroblastoma.

**Conclusions:**

Astroblastoma is a very rare primary brain tumor. Its diagnosis is often challenging because of the astroblastic aspects that can be found in astrocytic tumors, in ependymomas, and in non-neuroepithelial tumors. Considerable confusion surrounds its histogenesis and classification. The low incidence rate makes it difficult to conduct studies to examine tumor characteristics.

## Background

Astroblastoma is a controversial and an extremely rare central nervous system neoplasm [1–4]. It accounts for 0.45 to 2.8% of all neuroglial tumors and it is mainly located in the cerebral hemispheres of children, teenagers, and young adults [1, 2, 4–9]. It was initially described by Bailey and Cushing in 1926 [10] as a separate glial tumor and further characterized by Bailey and Bucy in 1930 [11]. Although its histogenesis has been clarified recently, controversies exist regarding its cellular origin and validity as a distinct entity, because it shares features of both astrocytomas and ependymomas [1, 5]. In the literature on brain tumor classification, this tumor has been categorized as follows: as a stage in the process of glioma dedifferentiation [12], as an astrocytoma of large cells producing fibers [13], or as a rare tumor, probably originating from tanycytes or ependymal astrocytes [14, 15]. Finally, it was listed among “other neuroepithelial tumors” in the *WHO Classification of Tumours of the Central Nervous System* [16]. Astroblastomas can be graded as either a low-grade or high-grade (anaplastic/malignant) variant. This histopathologic subtyping was applied by many pathologists, but has not yet been integrated in the World Health Organization (WHO) classification [3, 17]. Because of its extreme rarity and because its radiologic and histopathologic features are common and shared with other glial neoplasms, this tumor is prone to misdiagnosis and remains challenging not only in terms of diagnosis and classification but also in the subsequent management. The radiologic and histopathologic features help differentiate it from the more common ependymoma and astrocytoma [2, 4, 9].

This case report describes a new case of a young girl with astroblastoma. It discusses clinical, radiologic, pathological, and therapeutic features and the differential diagnosis of this rare neoplasm, with a large review of the literature.

## Case presentation

**Fig. 1:**
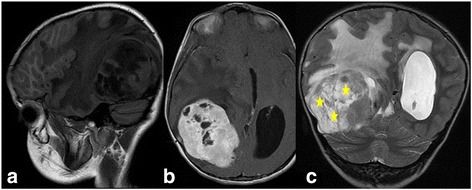
Cranial magnetic resonance imaging revealed a right, occipito-temporal, voluminous, well-demarcated mass, hypointense heterogenous on T1-weighted images (**a**) with a strong contrast enhancement (**b**) and a characteristic multicystic bubbly appearance on T2-weighted images (**c**). There was a peritumoral edema and a monoventricular left hydrocephaly. The stars indicate the tumor

**Fig. 2:**
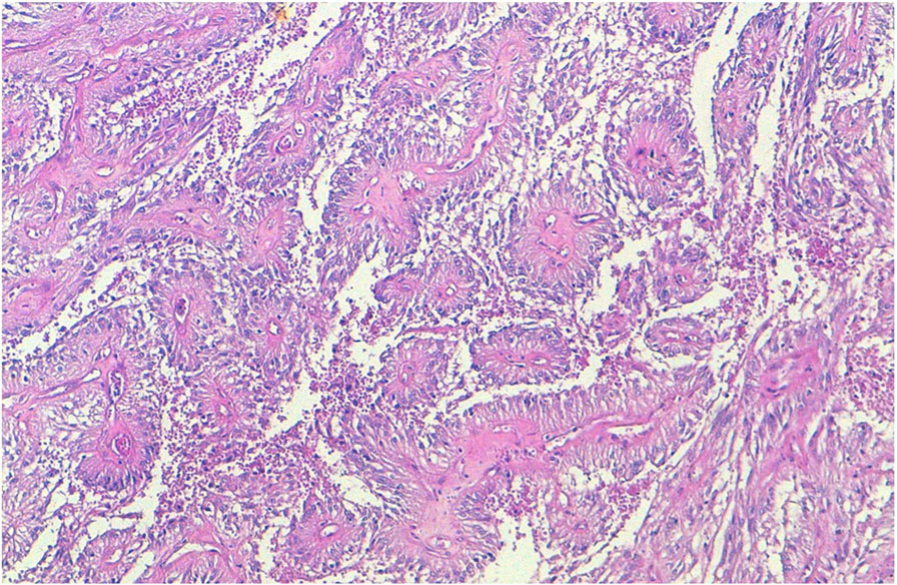
Microscopic appearance: tumor composed of perivascular rosettes of tumor cells (hematoxylin and eosin stain; original magnification × 100)

**Fig. 3:**
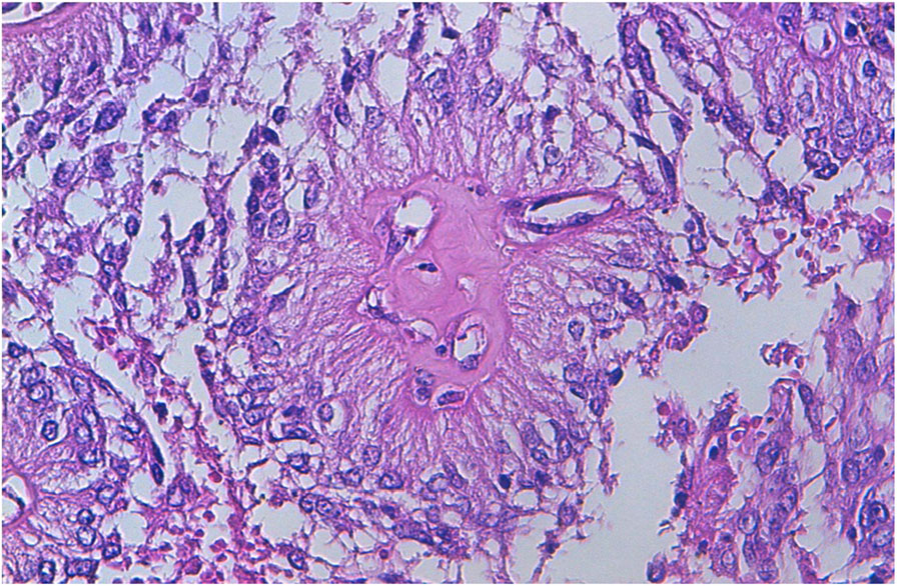
Microscopic appearance: Tumor cells with indistinct cytoplasmic borders and round to oval nuclei, without nuclear pleomorphism or mitotic activity (hematoxylin and eosin stain; original magnification × 400)

**Fig. 4:**
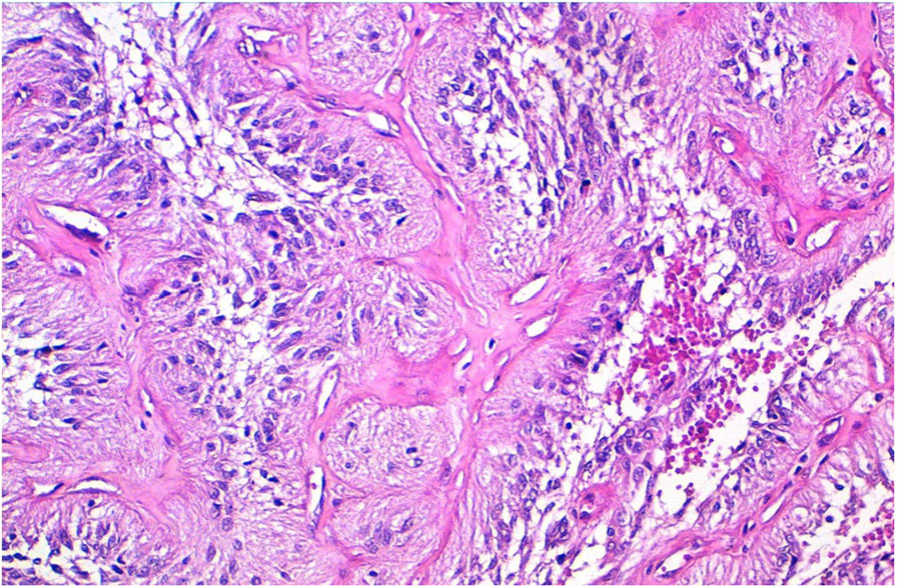
Microscopic appearance: thickened and focally hyalinized blood vessel walls (hematoxylin and eosin stain; original magnification × 100)

**Fig. 5:**
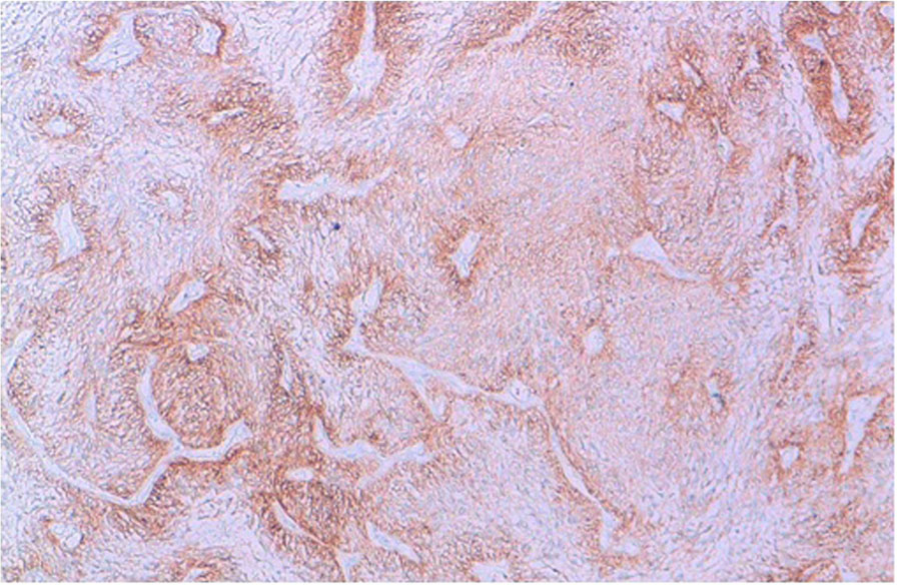
Positive immunostaining for glial fibrillary acid protein (original magnification × 100)

We report the case of an 8-year-old Moroccan girl who presented with a 1-year history of epileptic seizure, headache, and decreased visual acuity. Cranial magnetic resonance imaging (MRI) revealed a right, occipito-temporal, voluminous, well-demarcated mass with a multicystic component and a characteristic bubbly appearance on T2-weighted images and hypointense heterogenous on T1-weighted images. After contrast injection, a strong contrast enhancement was observed. There was a peritumoral edema and a monoventricular left hydrocephaly (Fig. 1). MRI findings suggested a diagnosis of a glial neoplasm. An incomplete (because of the hemorrhage) tumor resection was performed and microscopic examination revealed a tumor composed of perivascular rosettes of tumor cells (Fig. 2). The tumor cells had indistinct cytoplasmic borders. The nuclei were generally round to oval in shape, without nuclear pleomorphism or mitotic activity (Fig. 3). The tumor was very vascular with thickened and focally hyalinized blood vessel walls (Fig. 4). An immunohistochemical analysis showed positive staining for glial fibrillary acid protein (GFAP; Fig. 5). The cells were negative for epithelial membrane antigen (EMA) and Ki-67 labeling index was approximately 7%. P53 immunostaining was negative. Isocitrate dehydrogenase (*IDH*)*1*/*2* gene analysis by polymerase chain reaction (PCR) sequencing did not reveal mutation. Based on these data, the diagnosis of low-grade astroblastoma was confirmed.

## Discussion

Astroblastoma is one of the rarest central nervous system gliomas. It can occur in persons of any age, with a bimodal age distribution, with one peak in infancy (between 5 and 10 years) and the other one in young adults (between 21 and 30 years). The studies performed to date show a striking female preponderance with a male to female ratio of 1:11 [5–8, 18].

The tumor usually presents as a well-circumscribed and superficial mass, usually supratentorial with occipital and frontal lobes the most frequently affected sites. However, tumor invasion has also been reported into corpus callosum, cerebellum, brain stem, and optic nerve [1, 2, 6, 8].

Clinical signs and symptoms are dependent on the location and size of the tumor and primarily consist of those associated with elevated intracranial pressure. Headache, seizures, vomiting, and focal neurologic deficits are the most commonly mentioned symptoms [2, 4, 7].

Considerable confusion has surrounded the diagnosis, the histogenesis, and the classification of astroblastoma. Controversy still exists in the literature of the cell of origin of this neoplasm. Bailey and Bucy [11] believed that astroblastoma originated from astroblasts, an intermediate stage between glioblasts and astrocytes. However, Russell and Rubinstein [14] suggested that astroblastomas are dedifferentiated from mature astroglial cells. Later, in a study by Rubinstein and Herman [19], using electron microscopy, it was proven that astroblastomas might originate from persisting groups of embryonic precursor cells, transitional between astrocytes and ependymal cells. Given the lack of consensus, astroblastomas are currently classified as other neuroepithelial tumors by the WHO 2007 [16]; however, lack of sufficient clinicopathological data thwarts the WHO grading of these tumors [6].

On radiographic examination, the lesions show a characteristic appearance that may aid the pathologist in making the diagnosis of astroblastoma. On MRI, it is almost exclusively seen supratentorially and is peripheral in location. It typically appears as a large, well-demarcated, lobulated mass. It often has solid and cystic components with a characteristic bubbly appearance in the solid component, which was believed to result from the tumor vascular architecture, with inhomogeneous contract enhancement and little vasogenic edema [1, 2, 4–6, 8, 9, 18]. It is hyperintense to white matter on fast fluid-attenuated inversion recovery (FLAIR) images and T2-weighted images and hypointense to isointense on T1-weighted images [1]. Our case showed a typical solido-cystic lesion with a bubbly appearance.

On macroscopic examination, astroblastomas were described as superficial, well-demarcated, lobulated, solid, or cystic masses [4].

On histologic examination, an astroblastoma is defined by the presence of perivascular pseudorosettes and prominent perivascular hyalinization. The perivascular pseudorosettes give the characteristic “cartwheel” appearance. They exhibit characteristic epithelioid cells with cytoplasmic processes having blunt-ended foot plates attached to the basal lamina of blood vessels [2, 6, 8, 9]. The amount of perivascular hyaline formation varies from case to case; but in the most severe forms, expansive, acellular hyalinized zones will be seen without any residual tumor architecture [9]. Another feature of diagnostic importance is lack of fibrillary background [6]. Astroblastic features must be present in all the tumor extension to make the diagnosis of astroblastoma [8]. This tumor is mostly well circumscribed. Higher grade lesions will occasionally have clusters of tumor cells extending marginally into surrounding brain; however, there are no reports of diffuse infiltration of the surrounding tissue [9]. In our case, prominent hyalinization of the capillary network occurred only focally.

Because astroblastoma exhibits a highly variable biological behavior, a WHO grade has not been established yet. Based on morphology, Bonnin and Rubinstein [20] reported two distinct histological types: prognostically favorable “low-grade/well-differentiated” and unfavorable “high-grade/anaplastic” groups. The former includes astroblastomas with uniform perivascular arrangement of pseudorosettes, low to moderate numbers of mitotic figures, minimal cellular atypia, minimal to no vascular endothelial proliferation, and predominant sclerosis of the vascular walls. They are generally indolent and associated with a more favorable prognosis after surgical resections. The latter shows focal or multifocal regions of high cellularity, anaplastic nuclear features, high mitotic rates, vascular proliferation, and necrosis with pseudopalisading. They have shorter postoperative survival times. Our case was considered to be in the low-grade group as it had an orderly growth pattern with no evidence of necrosis, cellular atypia, high mitotic activity, or vascular endothelial proliferation.

Immunohistochemical features of astroblastoma have some variability throughout the literature. Immunostaining for GFAP is positive, lending support to the theory that the tumor cell is derived from an astrocyte cell line. Astroblasts also consistently stain positive with vimentin, suggesting derivation from a more primitive astroblast, and for S-100 protein. Other immunostains, such as neuron-specific enolase (NSE), EMA, cytokeratin (CK), and CAM 5.2, have had highly variable results in the current literature [2, 6, 9].

Apart from our case, 53 reported cases of astroblastoma are reviewed in this work. Epidemiological, clinical, radiologic, and immunohistochemical characteristics and grading are summarized in Table 1.

**Table 1:** Reviewed patients with astroblastoma (epidemiological, clinical, radiologic, immunohistochemical, and grading characteristics)

Reference	Age /Gender	Location	Symptoms	MRI	Immunohistochemistry								Grade
					EMA	VIM	GFAP	PS100	NF	CK	Ki67	P53	
Thiessen *et al*. (1998) [27]	5; F	PL											LG
Thiessen *et al*. (1998) [27]	51; F	FL	Seizures										LG
Thiessen *et al*. (1998) [27]	5; F	PL	Diplopia, headaches	Cystic, contrast-enhancing mass with a ring of contrast enhancement and a peritumoral edema									LG
Thiessen *et al*. (1998) [27]	16; F	POL	Headaches										HG
Thiessen* et al*. (1998) [27]	5; F	POL	Headaches										HG
Thiessen *et al*. (1998) [27]	1; F	FL											HG
Thiessen *et al*. (1998) [27]	5; M	TL											HG
Port *et al*. (2002) [5]	30; M	FL		Well-circumscribed, lobulated mass with cystic and bubbly solid components and intense heterogeneous enhancement									LG
Port* et al*. (2002) [5]	42; F	TL		Well-circumscribed, lobulated, mass with cystic and bubbly solid components and with calcifications									LG
Port *et al*. (2002) [5]	24; F			Well-circumscribed, lobulated, mass with cystic and bubbly solid components									LG
Port *et al*. (2002) [5]	5; F			Solid and cystic mass with intense heterogeneous enhancement of the solid portion and rim enhancement of the cystic portion									HG
Port *et al*. (2002) [5]	3; F	Supratentorial		Well-circumscribed, heterogeneous mass with bubbly appearance and heterogeneous enhancement of the solid component and with cystic changes									HG
Port *et al*. (2002) [5]	15; F	Corpus callosum		Solid and cystic mass with heterogeneous enhancement of the solid portion and rim enhancement around the cystic portion									HG
Sugita *et al*. (2002) [28]	33; F	FL	Seizures	Enhancing lesion mass	+	++	+	+		–	2%		LG
Cabrera-Zubizarreta *et al*. (2002) [29]	18; F	FL	Motor deficit, headaches, diplopia	Well-circumscribed heterogeneous mass with solid and cystic areas									LG
Kim *et al*. (2004) [30]	15; F	FL	Headaches, diplopia, nasal hemianopia	Well-demarcated mass with cystic changes and with inhomogeneous enhancement after an injection of gadolinium	+	++	++	++	–	–	8%	16%	LG
Caroli *et al*. (2004) [31]	30; M	TL	Coma	Tumor with inhomogeneous enhancement			++				8%		HG
Kaji *et al*. (2006) [32]	17; M	Frontal operculum	Diplopia, headaches, hemiparesis	Solid and cystic mass with homogeneous enhancement after gadolinium	++	++	++	++			5.6%		LG
Lau *et al*. (2006) [33]	21; F	Parietal	PL	Well-defined, lobulated, contrast-enhancing mass with cystic change	+		++	++			> 5%		LG
Miranda *et al*. (2006) [34]	42; F	FL	Headache, seizure	Well-defined solid cystic mass with a heterogenous contrast enhancement		+	+	+					
Hata *et al*. (2006) [35]	16; F	PL	Headaches	Cystic, well enhanced with gadolinium mass			++				2%		LG
Kubota *et al*. (2006) [36]	8; F	FPL	Headache, deterioration of consciousness level, motor weakness	Heterogeneously enhanced large circumscribed round mass with peritumoral edema	+	++	++		–	+	15.6%		HG
Alaraj *et al*. (2007) [8]	33; M	TL	Headache, nausea	Highly vascular hemorrhagic lesion with edema and heterogeneous contrast enhancement	–	++	++				15%		HG
Notarianni *et al*. (2008) [9]	20; F	Brainstem	Headaches, numbness, diplopia, blurred vision, ataxia	Well-circumscribed, contrast-enhancing cystic lesion	++	++	+	++	–	–	≈7%		?
Eom *et al*. (2008) [1]	20; F	TL	Headache	Isointense mass with bubble-like appearance, little peritumoral edema, cleft-like area and a strong heterogeneous contrast enhancement after contrast injection	++	++	++	++		++	16%	14.8%	HG?
Fathi *et al*. (2008) [37]	53; M	PL	Lethargy, headaches, impairment of memory and word finding, unsteady gait	Remarkably circumscribed, contrast-enhancing mass with perifocal edema	–	++	++	++	–	–	< 1%; 4% in the recurrent tumor		LG
Unal *et al*. (2008) [38]	4; M	FPL	Deficits of balance and difficulty with walking	Cystic mass with solid mural nodule, little l edema, and heterogeneous contrast enhancement	–	++	++	++		–			HG
Salvati *et al*. (2009) [39]	30; M	TL	Intracranial hypertension	Cystic circular lesion with inhomogeneous enhancement and very light edema			+	++			8%		HG
Salvati *et al*. (2009) [39]	27; F	POL	Seizure				+	++					LG
Salvati *et al*. (2009) [39]	39; F	TL	Aphasia				+	++					LG
Salvati *et al*. (2009) [39]	43; F	FL	Hemiparesis	Circular lesion with a ring-shaped contrast enhancement			+	++					LG
Salvati *et al*. (2009) [39]	33; M	Rolandic area	Hemiparesis				+	++					HG
Salvati *et al*. (2009) [39]	50; F	OL	Hemianopsia				+	++					HG
Kemerdere *et al*. (2009) [40]	6; F	FPL	Nausea, vomiting, loss of balance and falls, hemiparesis, facial nerve palsy	Prominently cystic mass containing solid parts with heterogeneous enhancement after gadolinium injection	++		++			++	7%		HG
Kemerdere *et al*. (2009) [40]	7; F	PL	Seizure	Solid mass with prominent gadolinium enhancement	++		++				5%		HG
Mastrangelo *et al*. (2010) [41]	21; F	FPL	Headaches, vomiting		++	++	++				30%		HG
Mastrangelo *et al*. (2010) [41]	12; F	TL	Headaches	Contrast-enhancing, well-defined lobulated mass, with little peritumoral edema and multiple cysts included in the solid component	–		+	–			5–10%		HG
Bergkåsa *et al*. (2011) [3]	50; F	FL	Seizures	Well-circumscribed mass with patchy contrast enhancement	–		++	++	++	–	10%		HG
Bhattacharjee *et al*. (2011) [42]	4; F	POL	Irregularity of the bone over the parietal region, headache	Solido-cystic lesion with heterogeneous enhancement after gadolinium injection and bone erosion	–	++	++	++			15%		HG
Agarwal *et al*. (2012) [2]	12; F	PL	Headache, diplopia	Well-demarcated mass with peripheral contrast enhancement	++	++							LG
Khosla *et al*. (2012) [43]	11; F	FPL	Headache, vomiting, blurring of vision, seizures	Well-defined solid cystic mass with a strong heterogenous contrast enhancement		+	+	+			4%		HG
Nasit and Trivedi (2013) [44]	10; F	FPL	Headaches, seizures	Lobulated well-defined solido-cystic lesion with thick and intense peripheral contrast enhancement and edema	–	++	++	++	–	–	0.5%		LG
De la Garma *et al*. (2014) [45]	9; F	FPL	Headaches, nausea, vomiting, hemiparesis, seizures, aphasia	Multicystic lesion with bubbly heterogenous pattern with minimal edema and a mixed solid and peripheral rim enhancement after contrast injection	+	++	++				≈40%		HG
Janz and Buhl (2014) [17]	16; F	POL	Headaches, nausea, dizziness, vomiting	Hypointense lesion in T1-WI with peritumoral edema and peripheral, heterogenous bubbly enhancement	++		+						LG with transition to HG
Janz and Buhl (2014) [17]	24; F	TL	Headaches, seizures	Partly cystic mass with rim contrast enhancement of media and limited perifocal edema		++	+	+			focally > 30%		HG
Singh *et al*. (2014) [6]	12; F	PL	Seizures, headache, vomiting	Well-defined hyperintense, contrast-enhancing cystic lesion with mural nodule		++	++	++					LG
Yao *et al*. (2015) [46]	36; M	OTL	Headaches, nausea, vomiting		–	+	+		–	–	< 2%		LG
Narayan *et al*. (2015) [47]	16, M	OTL	Headaches, vomiting	Heterogenous mass with solid and cystic components with perilesional edema and calcifications	–	+	+	+		–	2–4%	< 10%	LG
Barakat *et al*. (2016) [48]	40; M	FL	Headache, nausea, vomiting, confusion	Well-demarcated enhancing, partially cystic, partially calcified lesion	+	++	++	++					?
Singla *et al*. (2016) [49]	30; F	FL	Headaches, altered sensorium, weakness	Well-defined spherical lesion with edema and hemorrhage									LG
Singla *et al*. (2016) [49]	11; M	FPL	Seizures	Lobulated lesion heterogeneously enhanced with gadolinium									HG
Yuzawa *et al*. (2016) [50]	18, F		Headache, nausea, numbness in the face and upper limb	Well-circumscribed, solid, and cystic lesion with a bubbly appearance, little perilesional edema, and marked contrast enhancement	++	++	+	+		+	10.8%		LG
Yeo *et al*. (2016) [51]	35; M	Lateral ventricle	Limb numbness and weakness	Lobulated, heterogenous mass			++						LG
Our case	8; F	OTL	Seizure, headaches, decreased visual acuity	Well-demarcated mass with a multicystic component, bubbly appearance, a strong contrast enhancement after contrast injection, and peritumoral edema	–		+				7%	–	LG

– negative immunostaining, *+* focal immunostaining, *++* diffuse immunostaining, CK cytokeratin, EMA epithelial membrane antigen, *F* female, *FL* frontal lobe, *FPL* fronto-parietal lobe, GFAP glial fibrillary acid protein, *HG* high grade, *LG* low grade, *M* male, MRI magnetic resonance imaging, *NF neurofilament, OL* occipital lobe, *OTL* occipito-temporal lobe, *PL* parietal lobe, *POL* parieto-occipital lobe, *TL* temporal lobe, *VIM* vimentin

The diagnosis of astroblastoma is often difficult. In fact, astroblastic features are not unique to astroblastoma and can also be found in other tumors. Therefore, the combination of the radiologic and the histopathologic characteristics is necessary for making a correct diagnosis. The main differential diagnoses are ependymoma and angiocentric glioma [4, 8, 9, 18]. The distinguishing features between astroblastoma and ependymoma are shorter and broader cellular processes and hyalinized or even sclerosed blood vessels [1, 3, 8, 9]. Furthermore, between the pseudorosettes are rarified spaces, in contrast to the compact intravascular architecture of the ependymoma and the lack of fibrillarity in astroblastoma helps to distinguish its pseudorosettes from those found in ependymomas [9]. Ependymomas may show a similar immunohistochemical pattern, but GFAP immunoactivity in ependymomas is often more intense than that in astroblastomas [8]. The distinction with angiocentric glioma is not clear-cut, because this is an ill-defined tumor entity, characterized by perivascular distribution of bipolar and spindle cells, with mild pleomorphism, an infiltrative border, and lack of high-grade features. On immunohistochemical examination, it is typically positive with antibodies to GFAP, S-100 protein, and vimentin. A dot-like pattern of immunoreactivity to EMA has also been described [18, 21]. Gemistocytic astrocytomas and glioblastomas frequently contain focal areas of perivascular pseudorosettes. Therefore, the diagnosis of astroblastoma should be reserved for well-demarcated gliomas purely or mainly composed of the characteristic gliovascular structure described. The distinction between astroblastomas and nonglial papillary tumors such as papillary meningiomas and metastases from papillary tumors is aided by immunohistochemical features that show positive staining with glial markers such as GFAP and S-100 [8].

Data on the molecular genetics of astroblastoma are rare and only recently available from the literature. A study by Brat *et al*. [22] demonstrated that astroblastomas have characteristic chromosomal aberrations because they exhibit gain of chromosomes 19 and 20. These anomalies are different from those of the ependymomas or astrocytic tumors, suggesting that astroblastoma is a distinct entity rather than a variant of ependymoma [1, 4]. Other alterations noted were losses on 9q, 10, and X chromosome [22]. Shuangshoti *et al*. [23] found loss of heterozygosity at the D19S412 locus on 19q in a cerebral astroblastoma. More recently, an absence of *IDH 1*/*2* and *TP53* mutations, which are known to be involved in the development of low-grade gliomas, was shown in astroblastomas [24, 25]. In this case, there was not *IDH1* mutation. P53 immunostaining was negative.

Since astroblastomas are rare and tumor descriptions in the literature concern only individual cases or small collections of cases, optimal treatment protocols have not been established. Total resection is the best treatment. It provides excellent tumor control rates [1, 2]. Subtotal resection should be avoided, if possible [26]. The addition of adjuvant focal radiotherapy after subtotal resection does not appear to provide equivalent outcomes to gross total resection. Adjuvant therapy for high-grade and recurrent cases is recommended [4, 6, 26]. Regular follow-up is required even in low-grade variants due to unpredictable behavior. Favorable prognosis is almost invariably associated with well-circumscribed tumors which permit total resection of tumor in all grades [2].

Several investigators have found that astroblastoma prognosis may be predicted by the histology and extent of resection. The low-grade astroblastomas are thought to have a better prognosis than the high-grade ones. Their prognosis is similar to that of low-grade gliomas. High-grade astroblastoma prognosis corresponds to that of anaplastic astrocytomas and has been associated with recurrence and progression [1, 8]. Ahmed *et al*. [7] presented the largest series of patients with astroblastoma described in the literature (*n* = 239). They found that older age, supratentorial location, and treatment prior to 1990 were poor prognostic factors. In addition, they thought the reason why patients with cerebellar tumors had better prognosis was that they might have earlier signs of increased intracranial pressure, leading to a quicker diagnosis and subsequently more timely treatment than their supratentorial counterparts. Similarly, better prognosis for patients diagnosed after 1990 might be related to multiple factors like advances in diagnostics and therapeutic modalities, such as MRI [4].

## Conclusions

Astroblastoma is a very rare primary brain tumor. Its diagnosis is often challenging because of the astroblastic aspects that can be found in astrocytic tumors, in ependymomas, and in non-neuroepithelial tumors. Considerable confusion surrounds its histogenesis and classification. The low incidence rate makes it difficult to conduct studies to examine tumor characteristics.

## Abbreviations

CK: Cytokeratin
EMA: Epithelial membrane antigen
FLAIR: Fluid-attenuated inversion recovery
GFAP: Glial fibrillary acid protein
IDH: Isocitrate dehydrogenase
MRI: Magnetic resonance imaging
NSE: Neuron-specific enolase
PCR: Polymerase chain reaction
WHO: World Health Organization
